# Distinct B cell subsets give rise to antigen-specific antibody responses against SARS-CoV-2

**DOI:** 10.21203/rs.3.rs-80476/v1

**Published:** 2020-09-25

**Authors:** Christopher T. Stamper, Haley L. Dugan, Lei Li, Nicholas W. Asby, Peter J. Halfmann, Jenna J. Guthmiller, Nai-Ying Zheng, Min Huang, Olivia Stovicek, Jiaolong Wang, Maria Lucia Madariaga, Kumaran Shanmugarajah, Maud O. Jansen, Fatima Amanat, Isabelle Stewart, Siriruk Changrob, Henry A. Utset, Jun Huang, Christopher A. Nelson, Ya-Nan Dai, Paige D. Hall, Robert P. Jedrzejczak, Andrzej Joachimiak, Florian Krammer, Daved H. Fremont, Yoshihiro Kawaoka, Patrick C. Wilson

**Affiliations:** 1 Committee on Immunology, University of Chicago, Chicago, IL 60637, USA.; 2 University of Chicago Department of Medicine, Section of Rheumatology, Chicago, IL 60637, USA.; 3 Pritzker School of Molecular Engineering, University of Chicago, Chicago, IL 60637, USA.; 4 Influenza Research Institute, Department of Pathobiological Sciences, School of Veterinary Medicine, University of Wisconsin-Madison, Madison, WI 53711, USA.; 5 University of Chicago Department of Surgery, Chicago, IL 60637, USA.; 6 University of Chicago Department of Medicine, Chicago, IL 60637, USA.; 7 Department of Microbiology, Icahn School of Medicine at Mount Sinai, New York, NY 10029, USA.; 8 Department of Pathology and Immunology, Washington University School of Medicine, St Louis, MO 63130, USA.; 9 Center for Structural Genomics of Infectious Diseases, Consortium for Advanced Science and Engineering, University of Chicago, Chicago, IL 60637, USA.; 10 Structural Biology Center, X-ray Science Division, Argonne National Laboratory, Lemont, IL 60439, USA.; 11 Department of Biochemistry and Molecular Biology, University of Chicago, Chicago, IL 60637, USA.; 12 Division of Virology, Department of Microbiology and Immunology, Institute of Medical Science, University of Tokyo, 108-8639 Tokyo, Japan.; 13 These authors contributed equally.; 14 Lead Contact.

## Abstract

Discovery of durable memory B cell (MBC) subsets against neutralizing viral epitopes is critical for determining immune correlates of protection from SARS-CoV-2 infection. Here, we identified functionally distinct SARS-CoV-2-reactive B cell subsets by profiling the repertoire of convalescent COVID-19 patients using a high-throughput B cell sorting and sequencing platform. Utilizing barcoded SARS-CoV-2 antigen baits, we isolated thousands of B cells that segregated into discrete functional subsets specific for the spike, nucleocapsid protein (NP), and open reading frame (ORF) proteins 7a and 8. Spike-specific B cells were enriched in canonical MBC clusters, and monoclonal antibodies (mAbs) from these cells were potently neutralizing. By contrast, B cells specific to ORF8 and NP were enriched in naïve and innate-like clusters, and mAbs against these targets were exclusively non-neutralizing. Finally, we identified that B cell specificity, subset distribution, and affinity maturation were impacted by clinical features such as age, sex, and symptom duration. Together, our data provide a comprehensive tool for evaluating B cell immunity to SARS-CoV-2 infection or vaccination and highlight the complexity of the human B cell response to SARS-CoV-2.

## Introduction

Since the emergence of SARS-CoV-2 in December 2019, the World Health Organization has reported spread to over 200 countries with infections approaching 30 million and deaths 1 million worldwide. Despite this burden, the quest to identify effective vaccines, therapies, and protective biomarkers continues. The isolation of human monoclonal antibodies (mAbs) specific for immunogenic SARS-CoV-2 proteins holds immense potential, as they can be rapidly employed as therapeutic agents, diagnostic reagents, and aid vaccine optimization. Several independent groups have identified potently neutralizing mAbs against the SARS-CoV-2 spike protein, the major immunogenic surface glycoprotein^1–7^. Despite these advances, there have been no mAbs isolated against other immunogenic targets of SARS-CoV-2, including the internal nucleoprotein (NP) and open reading frame (ORF) proteins 7 and 8, which have been suggested to induce antibody responses and immunomodulatory effects in humans^8–12^. Moreover, the properties and frequencies of B cell subsets targeting distinct SARS-CoV-2 antigens remain poorly understood, and are likely shaped by clinical features such as age and disease severity^6,13,14^.

To address these knowledge gaps, we comprehensively characterized the SARS-CoV-2-specific B cell repertoire in convalescent COVID-19 patients and generated mAbs against the spike, ORF8, and NP proteins. Together, our data reveal key insight into antigen specificity and B cell subset distribution upon SARS-CoV-2 infection in the context of age, sex, and disease severity.

## Results

### SARS-CoV-2-specific B cell sequencing

**Fig. 1:**
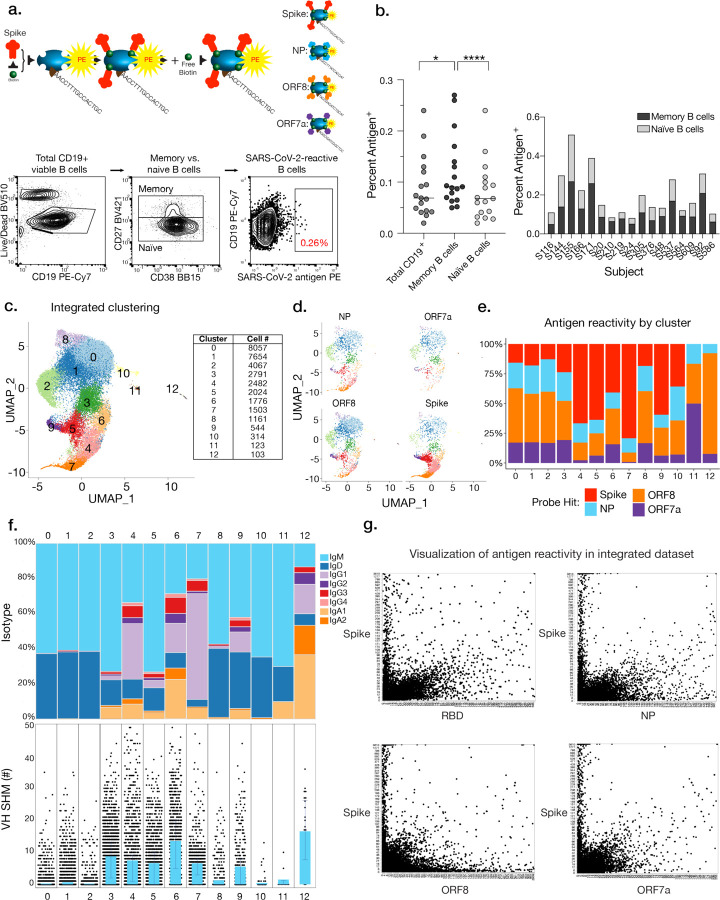
B cell subsets enriched for SARS-CoV-2-reactivity are revealed by transcriptome, Ig repertoire, and probe binding. **a**, Model demonstrating antigen probe preparation and representative gating strategy for sorting antigen-positive B cells. **b**, Percentage of antigen-probe-positive total B cells (CD19^+^CD3^−^), naïve B cells (CD27^+^CD37^int^), and memory B cells (CD27^+^CD38^int^) (left), and naïve vs. memory B cells by subject (right; n=17 subjects). Statistics are paired non-parametric Friedman test (*p=0.0491; ****p<0.0001). **c**, Integrated transcriptional UMAP analysis of distinct B cell clusters and the corresponding number of B cells per cluster. **d**, Feature library enrichment of antigen-probe-positive B cells by cluster. **e**, Percent probe reactivity of all B cells by cluster. **f**, Ig isotype usage and VH gene SHM for all antigen-positive B cells per cluster. Bars indicate median with interquartile range. **g**, Representative visualization of antigen reactivity revealing antigen-specific B cells. Axes indicate antigen probe intensities.

Serum antibodies and MBCs have potential to act as the first line of defense against SARS-CoV-2 infection^11,15–17^. To determine the landscape of antibody reactivity toward distinct SARS-CoV-2 viral targets, we collected peripheral blood mononuclear cells (PBMCs) and serum from 25 subjects between April and May of 2020 upon recovery from SARS-CoV-2 viral infection (Extended Data Table 1 and Extended Data Table 2). To identify B cells specific to the SARS-CoV-2 spike protein, spike RBD, ORF7a, ORF8, and NP, we generated probes to bait-sort enriched B cells for subsequent single cell RNA sequencing analysis by conjugating distinct phycoerythrin (PE)-streptavidin (SA)-oligos to individual biotinylated antigens (Fig. 1a).

**Extended Data Fig. 1.**
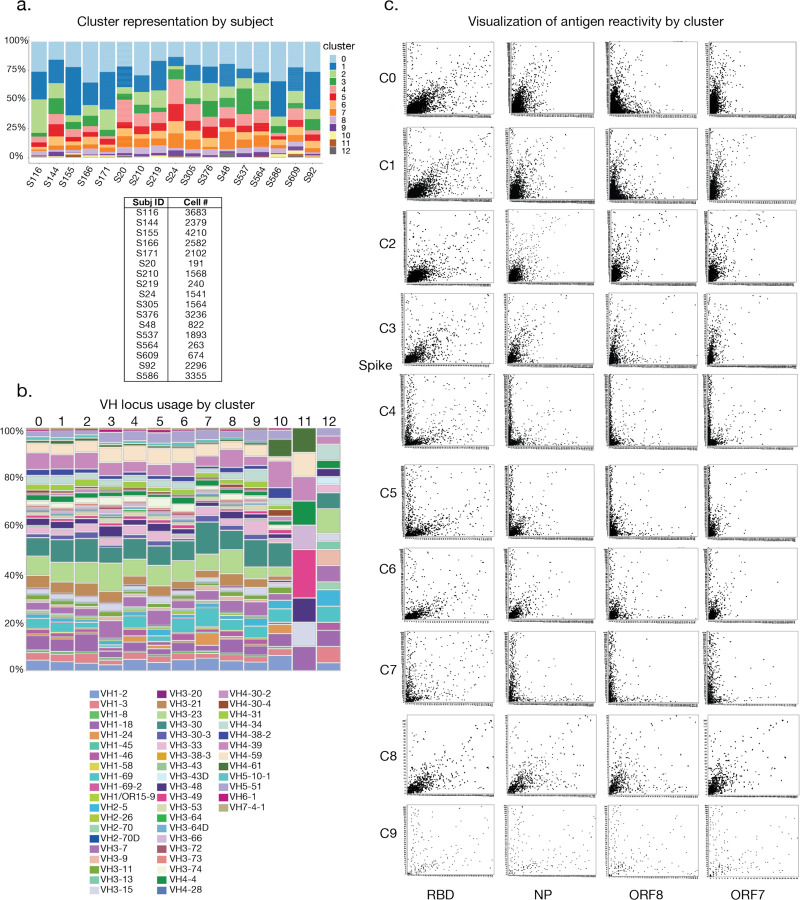
Additional characteristics of B cells comprising integrated clusters. **a**, Antigen-probe-positive B cell distribution across integrated clusters by subject with the number of cells per subject indicated. **b**, Variable gene segment usage in B cell receptor heavy chains of antigen-probe-positive B cells across integrated clusters. **c**, Diagrams showing antigen-probe-positive B cells per cluster with probe intensities for the indicated antigens plotted on the axes.

From 25 subjects analyzed, we detected small percentages (0.02–0.26%) of SARS-CoV-2-reactive total CD19^+^ B cells, which were subsequently used to prepare 5’ transcriptome, immunoglobulin (Ig) VDJ, and antigen-specific probe feature libraries for sequencing (Fig. 1a, b). We detected increased percentages of antigen-specific B cells within the memory B cell (MBC) compartment (Fig. 1b, CD19^+^CD27^+^CD38^int^), though we sorted on total CD19^+^ antigen-specific B cells to ensure adequate coverage of all potential reactive B cells and to optimize sequence library preparation and downstream analysis as the antigen-specific population was rare. We integrated data from 17 subjects with high-quality sequencing results using Seurat to remove batch effects and identified 12 transcriptionally distinct B cell clusters based on transcriptional expression profiles (Fig. 1c). It was immediately evident that B cells specific to the spike, NP, and ORF8 were found amongst multiple B cell subsets, with spike-specific B cells substantially enriched in clusters 4, 5, 7, and 9 (Fig. 1d, e). Analysis of Ig isotypes and degree of Ig variable heavy chain somatic hypermutations (VH SHM) suggested that clusters 0–2, 8, 10, and 11 represented naïve- or innate-like B cell clusters predominantly composed of IgM and IgD B cells. In contrast, clusters 3, 4, 5, 6, 7, 9, and 12 strongly indicated B cell subsets more similar to MBCs or plasma cells, as they exhibited a higher degree of class switch recombination (CSR) and/or increased numbers of VH SHM (Fig. 1f). We detected variation in the percentage of total cells sorted per cluster amongst individual patients, reflecting differences in the biology of individual responses to SARS-CoV-2, as we expand upon later (Extended Data Fig. 1a). No major differences in VH gene usage across clusters were evident, though we identified enrichment of VH1–24 in cluster 7, which we later identify as exclusively utilized by spike-reactive B cells (Extended Data Fig. 1b).

We next addressed whether the probe intensities generated from our feature libraries correlated with antigen-specific reactivity by plotting intensities for distinct probes against one another to observe true specificity (cells that fall directly onto the x or y axis) vs. non-specific binding (cells that fall on the diagonal). We observed hundreds of cells specific to the spike, ORF8, and NP, and to a lesser degree, the RBD alone and ORF7a (Fig. 1g). For clusters 0, 1, 2, and 8, we observed that the majority of cells were not uniquely specific for any one probe, and instead tended to bind more than one probe in a polyreactive or non-specific manner, consistent with innate-like B cells^18^. Finally, clusters 4, 5, 6, 7, and 9 exhibited highly specific binding toward the spike, NP, and ORF8, with the majority targeting the spike (Extended Data Fig. 1c). Together, our data suggest the B cell response to SARS-CoV-2 is comprised of multiple functionally distinct B cell subsets enriched for binding to distinct viral targets.

### SARS-CoV-2-specific B cell subsets

**Fig. 2:**
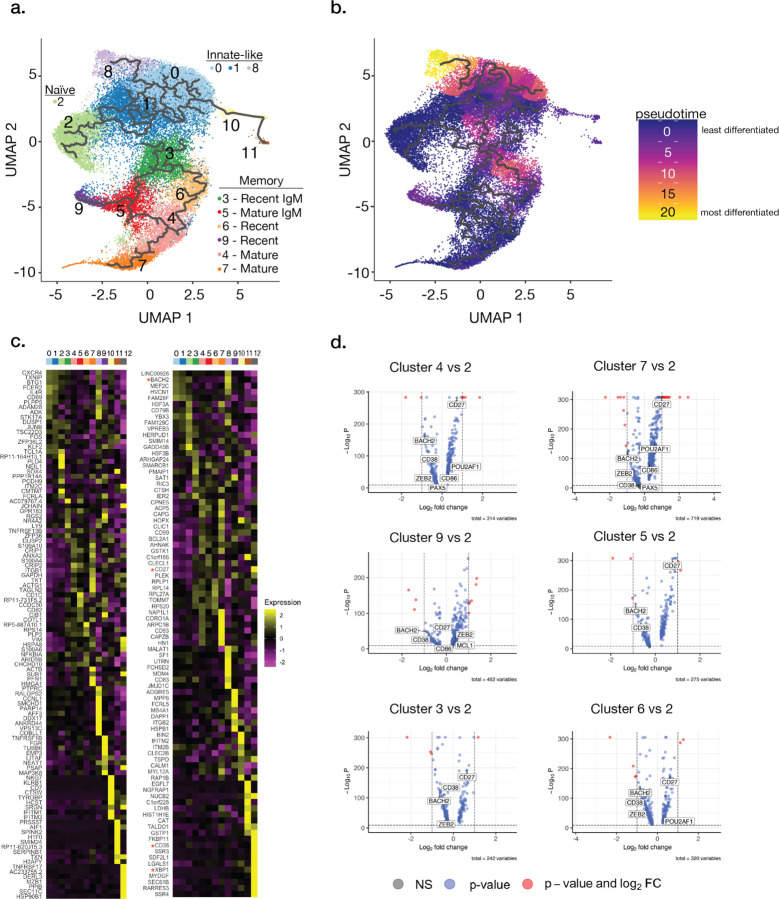
Transcriptional analysis distinguishes naïve, innate-like and MBC subsets specific to SARS-CoV-2 proteins. **a–b**, Trajectory (**a**) and pseudotime (**b**) analyses of clusters 0–11 reveals least to most differentiated clusters. Cluster 12 is excluded from trajectory analysis as it represents a separate partition as defined by Monocle3. **c**, Heatmap showing the top twenty most differentially expressed genes per cluster. Red stars denote genes used in memory B cell (MBC) identification. **d**, Volcano plots comparing differentially expressed genes in MBC-like clusters relative to cluster 2 (naïve B cells). Genes used in MBC identification are indicated: *cd27*, *cd38, hhex, zeb2, pou2af1*, *spib*, *cd80*, *cd86*, *mcl1, prdm1, abp1, manf, bach2, pax5*. Red-colored dots represent a log fold change in expression >0.1 and an adj-p value <0.01. Putative B cell subset identities are highlighted where they could be clearly defined (**a**).

To discern the identities of distinct B cell subsets, we further analyzed Ig repertoire, differentially expressed genes, and performed pseudotime analyses of integrated clusters. For pseudotime analysis, we rooted the data on cluster 2, as cells within this cluster expressed Ig genes with little to no SHM or CSR (Fig. 1f) and displayed low probe reactivity (Extended Data Fig. 1c), suggesting this subset is comprised of true naïve B cells. Pseudotime analysis rooted on cluster 2 identified clusters 0, 1, and 8 in various stages of differentiation, suggestive of recent activation (Fig. 2a–b). As they displayed little CSR or SHM (Fig. 1f), we therefore categorized these subsets as innate-like or possibly germinal center independent. Clusters 3 and 5 appeared to be specific IgM memory subsets (Fig. 1f and Extended Data Fig. 1c), while clusters 4, 7, 9, and 12 displayed high specificity, CSR, and SHM, demonstrating an affinity-matured memory phenotype (Fig. 1f and Extended Data Fig. 1c). As naïve B cells and MBCs are quiescent, clusters 4, 5, 7, and 9 were similar to cluster 2 in pseudotime analysis (Fig. 2a–b)^19^. Lastly, cluster 6 was of interest as these cells displayed the greatest frequency of SHM and IgA CSR, and may have arisen in the context of a mucosal immune response.

**Extended Data Fig. 2.**
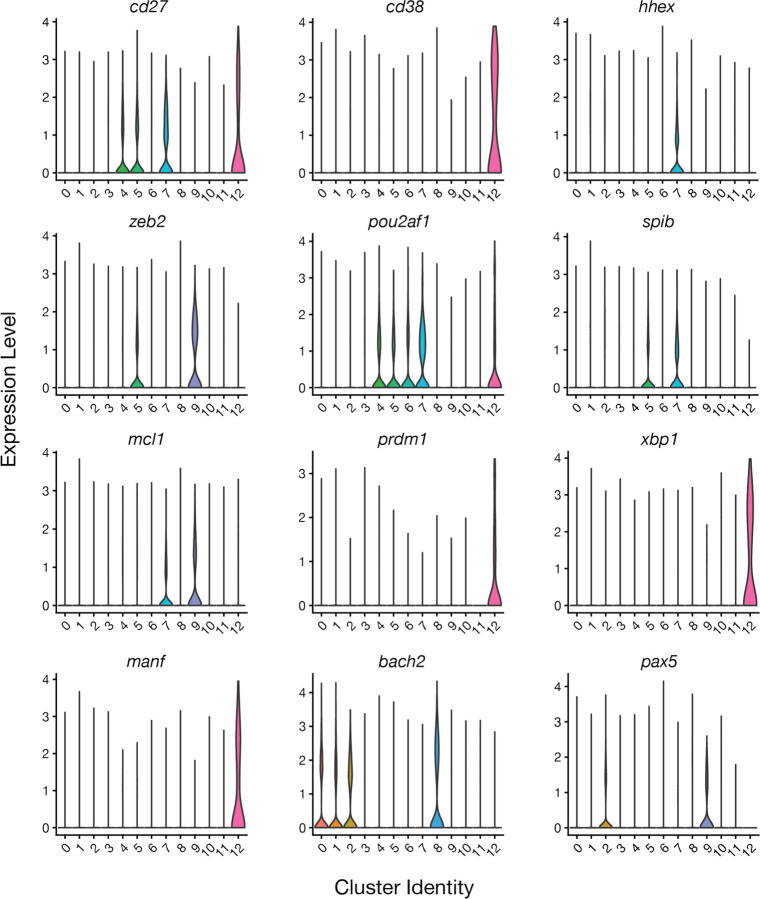
Expression of MBC and LLPC gene markers in integrated clusters. Normalized expression levels of the indicated genes represented as violin plots.

In-depth analysis of select genes including those related to B cell fate, MBC differentiation and maintenance, and long-lived plasma cells (LLPCs) helped to further reveal the identities of select clusters. Genes associated with MBCs (*cd27*, *cd38*, *cd86*, *pou2af*), repression of apoptosis (*mcl1)*, early commitment to B cell fate (*zeb2*), repression of LLPC fate (*spiB, pax5, bach2*), and early B cell activation and proliferation (*bach2*) confirmed clusters 3, 4, 5, 7 and 9 as MBCs though with varying degrees of differentiation, CSR, and SHM (Fig 2b–c and Extended Data Fig 2). Notably, we identified upregulation of the transcription factor *hhex* in cluster 7, which has recently been shown to be involved in MBC differentiation in mice (Extended Data Fig. 2)^20^. Lastly, cluster 12 appeared to be LLPCs or precursors thereof by expression of genes associated with LLPC fate, including *prdm1*, *xbp1*, and *manf* (Extended Data Fig. 2)^19,21,22^. Together with our antigen-specific probe data (Fig. 1), these results confirm that clusters representing classical MBCs are enriched for spike binding while B cells targeting internal proteins are enriched in activated naïve and innate-like B cell subsets.

### SARS-CoV-2-specific Ig repertoire

**Extended Data Fig. 3.**
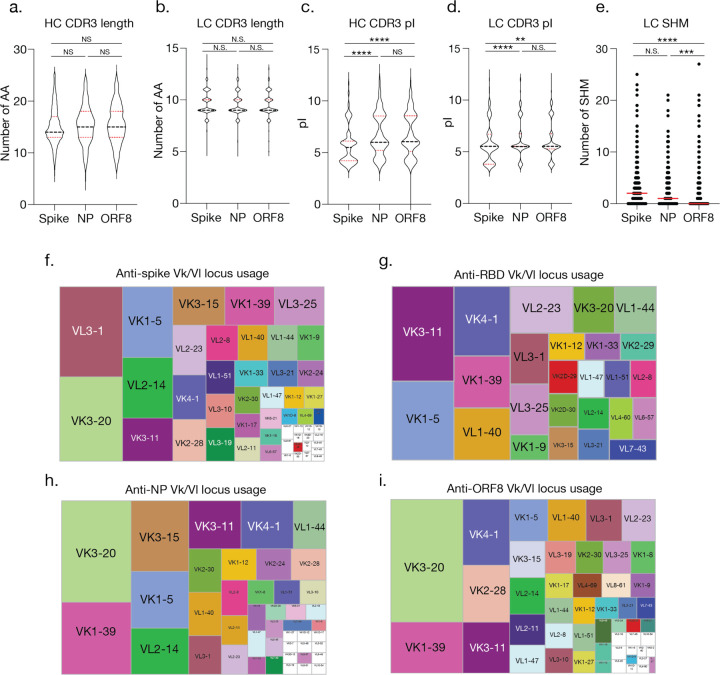
Heavy and light chain features of SARS-CoV-2 reactive B cells. **a–b,** Heavy chain (HC, **a**) and light chain (LC, **b**) complementarity determining region 3 (CDR3) lengths, shown by antigen-reactivity. **c–d,** HC (**c**) and LC (**d**) isoelectric points pI, shown by antigen-reactivity. **e,** Number of light chain (LC) somatic hypermutations (SHM), shown by antigen-reactivity. **f–i.** Tree maps showing frequency of Vk/L gene locus usage for spike- (**f**), RBD- (**g**), NP- (**h**), and ORF8-specific B cells (**i**). In panels **a–e** groups were compared by Kruskal-Wallis test (N.S.= not significant, ****p<0.0001; ***p=0.0006; **p=0.0033). For **f–i**, n=531 for spike, n=47 for RBD, n=293 for NP, and n=463 cells selected for ORF8.

**Fig. 3:**
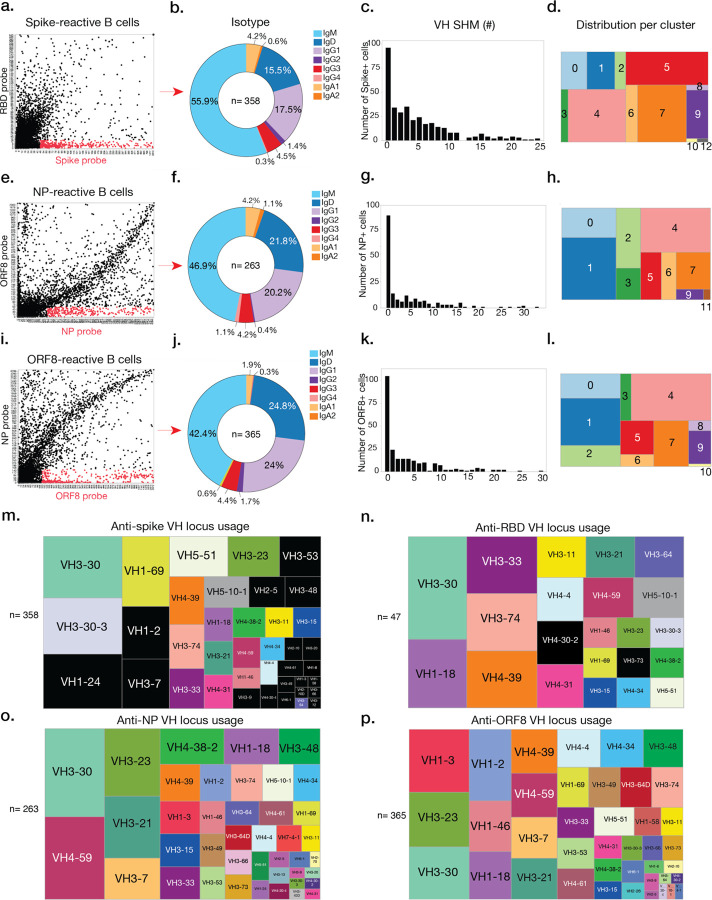
SARS-CoV-2-reactive B cells exhibit unique features for isotype, SHM, subset of origin, and VH gene usage. **a–l**, Ig isotype, VH gene SHM, and distribution of B cells by integrated cluster for spike-(**a, b, c, d**), NP-(**e, f, g, h**) and ORF8-specific B cells (**i, j, k, l**). **m–p**, Tree maps showing frequency of VH gene locus usage for total spike (including RBD) (**m**), RBD only (**n**), NP (**o**), and ORF8-specific B cells (**p**). Numbers in the center of each pie chart and below each tree map indicate number of cells analyzed per reactivity.

The properties of B cells targeting immunogenic targets such as ORF8 and NP compared to the spike are unknown. We further analyzed isotype frequencies, VH SHM, VH gene usages, and frequencies of B cells against these targets within distinct B cell subsets. The majority of antigen-specific B cells were of the IgM isotype with a limited degree of CSR. There were no major differences between the isotypes of B cells specific to these distinct targets, with the majority of class-switched cells being of the IgG1 isotype. Consistent with a *de novo* response against the novel SARS-CoV-2, we observed that the majority of antigen-specific B cells had little to no VH SHM, though spike-reactive B cells displayed slightly increased amounts of SHM. Spike-specific B cells were primarily enriched in MBC and LLPC-like clusters 4, 5, 7, 9, and 12 while NP- and ORF8-specific B cells were largely found within naïve- and innate-like clusters but also within MBC clusters (Fig. 3a–l). Lastly, we did not observe differences in heavy chain (HC) or light chain (LC) complementarity determining region 3 length by antigen targeting (Extended Data Fig. 3a–b), though we did observe that HC and LC isoelectric points (pI) for spike-reactive B cells were generally lower than NP- or ORF8-reactive B cells (Extended Data Fig. 3c–d), and LC SHM was greater for spike-reactive B cells (Extended Data Fig. 3e).

We next analyzed the VH gene usages of spike-, NP-, and ORF8-specific B cells and identified the most common VH usages per reactivity (represented by larger squares on each tree map) as well as shared VH usages across reactivities (shown by matching colors; Fig. 3m–p). Strikingly, we identified usage of particular VH gene loci that did not overlap between spike- and RBD-reactive B cells (shown in black). VH1–24, VH3–7, and VH3–9 were the highest represented VH gene usages exclusively associated with non-RBD spike reactivity, and VH1–24 usage was enriched in cluster 7, an MBC-like cluster (Fig. 3m–n and Extended Data Fig. 1b). These results were confirmed by mAb data, which identified spike-specific mAbs utilizing VH1–24 and VH3–7 that did not bind to the RBD (Extended Data Table 3). Unique LC V gene usages were also evident amongst antigen-specific cells (Extended Data Fig. 3f–i).

Finally, public B cell clones were of interest as the epitopes bound can be targeted by multiple people and thus represent important vaccine targets. We identified five novel public clones from this dataset, three of which were present in two separate subjects, one that was present amongst three subjects, and one amongst four subjects (Extended Data Table 4). Four of the clonal pools were specific to the spike protein, and the remaining clone to NP. The majority of clonal pool members were identified in MBC-like clusters 3, 4, 5, 7, and 9, suggesting that B cells specific to public epitopes can be established within stable MBC compartments.

### Monoclonal antibody binding and neutralization

**Extended Data Fig. 4.**
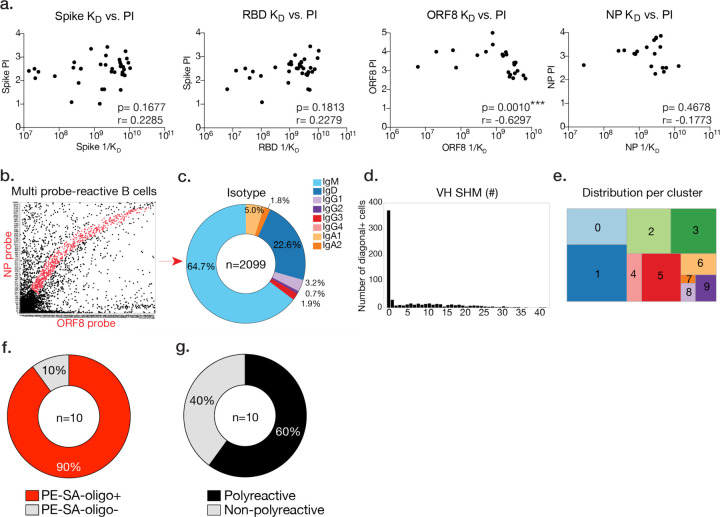
Additional features of mAbs cloned from antigen-specific and multi-probe binding B cells. **a**, ELISA K_D_ for specific mAbs against the spike, RBD, ORF8, and NP, versus normalized probe intensity for spike, ORF8, and NP respectively. Whole spike antigen probe intensities are plotted for RBD-binding mAbs. Statistics are Spearman correlations with p and r values indicated. **b**, Example selection of multi-probe-reactive B cells. **c**, Isotype frequencies of multi-probe-reactive B cells. **d**, Number of VH gene SHM for multi-probe-reactive B cells. **e**, Proportion of multi-probe-reactive B cells in integrated clusters. **f**, Percentage of multi-probe-reactive B cells binding PE-SA-oligo by ELISA. **g**, Percent multi-probe-reactive B cells exhibiting polyreactivity, as determined by ELISA. Numbers in the center of each pie chart indicate number of B cells/mAbs analyzed.

**Fig. 4:**
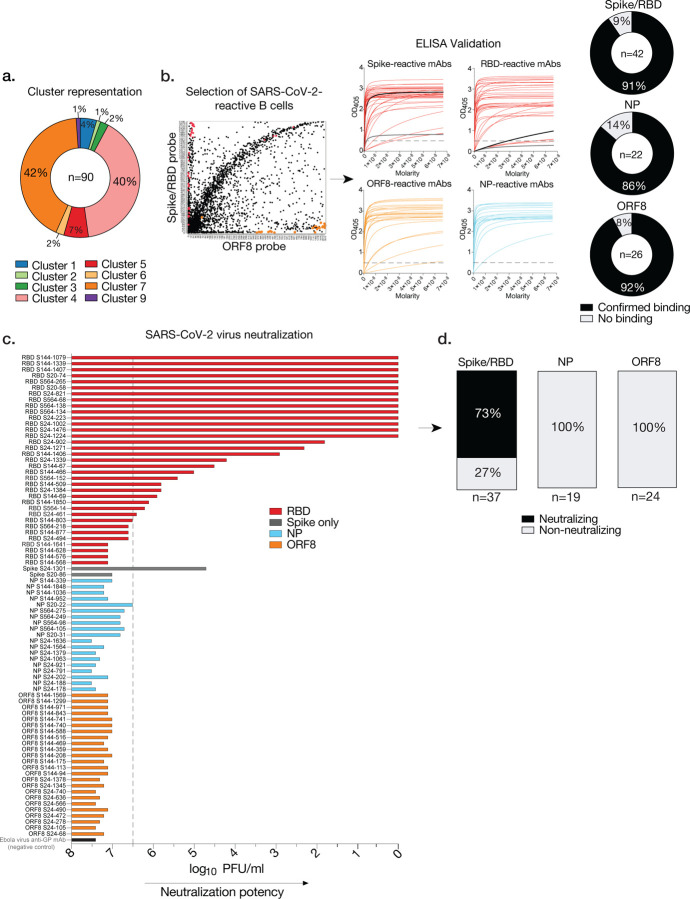
Characterization of mAbs from single SARS-CoV-2-reactive B cells. **a**, Cluster origin of cloned mAbs (n=90). **b**, Representative plot showing the selection of B cells chosen to clone mAbs, antigen binding curves by ELISA for each reactive mAb (spike, n= 38; RBD, n=36; NP, n=19; ORF8, n=24), and percentages of total cloned mAbs exhibiting specificity (right). Dashed line on ELISA curves represents the OD_405_ cutoff of 0.5 for positivity. **c**, Neutralization potency (log_10_ PFU/ml) of mAbs tested by live SARS-CoV-2 virus plaque assay. Dashed line at x= 6.5 indicates cutoff for neutralization. **d**, Percentage of total spike, NP, and ORF8-specific mAbs that displayed neutralization activity. Numbers below each bar chart indicate the number of mAbs tested for neutralization. ELISA data are representative of 2–3 idependent experiments and mAbs were screened once for neutralization ability.

To simultaneously validate the specificity of our approach and investigate the properties of mAbs targeting distinct SARS-CoV-2 viral epitopes, we synthesized and characterized the binding and neutralization ability of 90 mAbs from our single cell dataset (Extended Data Table 3). B cells exhibiting variable probe binding intensities toward distinct antigens were chosen as candidates for mAb generation, as well as B cells that tended to bind multiple probes (exhibiting non-specificity or polyreactivity). MAbs cloned were representative of various clusters, reactivities, VH gene usages, mutational load, and isotype usages (Fig. 4a, Extended Data Table 3). Representative mAbs generated from cells specific to the spike, NP, and ORF8 exhibited high affinity by ELISA, though probe intensities did not meaningfully correlate with apparent affinity (K_D_) (Fig. 4b, Extended Data Fig. 4a). Only a small percentage of cloned mAbs to the spike, NP, and ORF8 exhibited non-specific binding (Fig. 4b). Notably, cells exhibiting non-specific binding were reactive to the PE-SA-oligo probe conjugate and were largely polyreactive (Extended Data Fig. 4b–g).

While mAbs targeting the RBD of the spike are typically neutralizing, little is known regarding the neutralization capabilities of mAbs targeting non-RBD regions of the spike, ORF8 and NP. We addressed the neutralization ability of all synthesized mAbs using a live virus plaque assay and determined that all mAbs cloned against NP and ORF8 were non-neutralizing, while mAbs against the RBD and other epitopes of the spike were largely neutralizing at varying degrees of potency (Fig. 4c–d). As anti-spike mAbs were predominantly neutralizing and enriched in memory, these MBC subsets may serve as a biomarker for superior immunity to SARS-CoV-2.

### Antigen targeting and clinical features

**Fig. 5:**
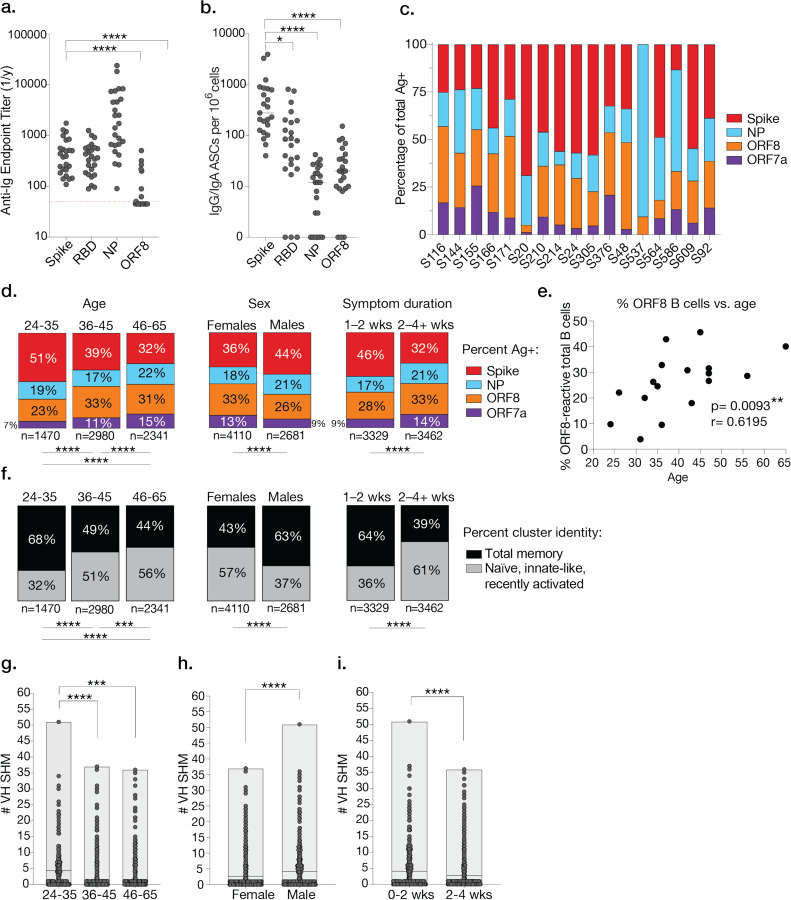
B cell antigen targeting, subset distribution, and adaptability is linked to clinical features. **a,** Total serum anti-Ig endpoint titers for SARS-CoV-2 antigens determined by ELISA (n=25 subjects). **b,** Number of IgG/IgA antibody secreting cells (ASCs) per 10^6^ cells determined by ELISpot (n=23 subjects). **c.** Percentage of antigen-probe-positive cells by subject. **d,** Percentage of antigen-probe-positive cells stratified by age (in years), sex, and symptom duration (in weeks). **e,** Spearman correlation between percentage of all cells specific to ORF8 and subject age with p and r values indicated. **f,** Percentage of antigen probe positive B cells in MBC-like clusters (3, 4, 5, 6, 7, 9, and 12) or naïve and innate-like clusters (0, 1, 2, 8, 10, 11) stratified by age, sex, and symptom duration. **(g–i)** VH gene SHM for antigen-specific cells from a given age (**g**), sex (**h**), or symptom duration group (**i**). Data in **a** and **b** were analyzed using paired non-parametric Friedman tests with multiple comparisons against the spike (*p=0.0154, ****p<0.0001). Red dashed line in **a** at y=45 indicates cutoff for no serum titer detected. The data in **d** and **f** were analyzed using Chi-square or Fisher’s exact tests, (****p<0.0001; ***p=0.0009). Data in **g** were analyzed using unpaired non-parametric Kruskal Wallis (****p<0.0001; ***p=0.0002). Statistics used in **h** and **i** are unpaired non-parametric Mann-Whitney tests (****p<0.0001).

Previous studies from our group and others have suggested serum antibody titers correlate with sex, SARS-CoV-2 severity, and age^6,14,23^. We therefore investigated the frequencies of SARS-CoV-2-reactive B cells to assess whether reactivity toward particular SARS-CoV-2 antigens correlated with clinical parameters. By both serology and ELISpot, we identified that B cell responses against the spike/RBD and NP were immunodominant, though ORF8 antigen targeting was substantial (Fig. 5a, b). Consistent with our single cell dataset, spike-specific B cells were enriched in memory by ELISpot (Fig. 5b).

We next analyzed the distribution of B cell subsets and frequencies of B cells specific to the spike, NP, ORF7a, and ORF8 in sets of patients stratified by age, sex, and duration of symptoms from our single cell dataset. We normalized antigen probe signals by a centered log-ratio transformation individually for each subject; all B cells were clustered into multiple probe hit groups according to their normalized probe signals, and cells that were negative to all probes or positive to all probes (non-specific) were excluded from the analysis. We identified substantial variation amongst individual subjects in terms of the degree of spike, NP, ORF7a, and ORF8 antigen targeting (Fig. 5c). As subject age increased, the percentages of spike-reactive B cells relative to B cells targeting internal proteins decreased, and age positively correlated with increased percentages of ORF8-reactive B cells (Fig. 5d–e). Similarly, female subjects and subjects experiencing a longer duration of symptoms displayed reduced spike targeting relative to internal proteins (Fig. 5d). Consistent with spike-reactive B cells enriched in MBC clusters, patient who were younger, male, or experienced a shorter duration of symptoms exhibited increased targeting of the spike and increased proportions of MBC subsets (Fig. 5d, f). Accordingly, older patients, female patients, and patients with a longer duration of symptoms exhibited reduced levels of VH gene SHM (Fig. 5g–i).

In summary, our study highlights the diversity of B cell subsets expanded upon novel infection with SARS-CoV-2. Using this approach, we identified that B cells against the spike, ORF8, and NP differ in their ability to neutralize, derive from functionally distinct and differentially adapted B cell subsets, and correlate with clinical parameters such as age, sex, and symptom duration.

## Discussion

The COVID-19 pandemic continues to pose one of the greatest public health and policy challenges in modern history, and robust data on long-term immunity is critically needed to evaluate future decisions regarding COVID-19 responses. Our approach combines three powerful aspects of B cell biology to address human immunity to SARS-CoV-2: B cell transcriptome, Ig sequencing, and recombinant mAb characterization. Our approach enables the identification of potently neutralizing antibodies and the characteristics of the B cells that generate them. Importantly, we showed that antibodies targeting key protective spike epitopes are enriched within canonical MBC populations.

Identification of multiple distinct subsets of innate-like B cells, MBCs, and apparent LLPC precursors illustrates the complexity of the B cell response to SARS-CoV-2, revealing an important feature of the immune response against a novel pathogen. The B cell clusters herein may provide biomarkers in the form of distinct B cell populations that can be used to evaluate future responses to various vaccine formulations. In particular, the identification of LLPC precursors in the blood following infection and vaccination has been long sought after, as they serve as a bonafide marker of long-lived immunity^24,25^. Future studies elucidating distinct identities and functions of these subsets are necessary and will provide key insights into B cell immunology.

We identified that older patients, female patients, and patients experiencing a longer duration of symptoms tended to display reduced proportions of MBC clusters and reduced VH SHM, consistent with a previous study that identified limited germinal center formation upon SARS-CoV-2 infection^26^. Notably, older patients had increased percentages of ORF8-specific B cells, which we identified as exclusively non-neutralizing. Mechanistically, these observations may be explained by reduced adaptability of B cells or increased reliance on CD4 T cell help for B cell activation, which have been observed in aged individuals upon viral infections^27,28^. Furthermore, T cell responses to SARS-CoV-2 ORF proteins are prevalent in convalescent COVID-19 patients, and recent studies suggest impaired T cell responses in aged COVID-19 patients impact antibody responses^10,29,30,42^. More research is warranted to definitively determine whether B cell targeting of distinct SARS-CoV-2 antigens correlates with age and disease severity. Addressing these questions will be critical for determining correlates of protection and developing a vaccine capable of protecting our most vulnerable populations.

## Materials & Methods

### Study cohort and sample collection

All studies were performed with the approval of the University of Chicago institutional review board IRB20–0523 and University of Wisconsin-Madison institutional biosafety committees. Informed consent was obtained after the research applications and possible consequences of the studies were disclosed to study subjects. This clinical trial was registered at ClinicalTrials.gov with identifier NCT04340050, and clinical information for patients included in the study is detailed in Extended Data Table 1 and Extended Data Table 2. Leukoreduction filter donors were 18 years of age or older, eligible to donate blood as per standard University of Chicago Medicine Blood Donation Center guidelines, had a documented COVID-19 polymerase chain reaction (PCR) positive test, and complete resolution of symptoms at least 28 days prior to donation. PBMCs were collected from leukoreduction filters within 2 hours post-collection and flushed from the filters using sterile 1X Phosphate-Buffered Saline (PBS, Gibco) supplemented with 0.2% Bovine Serum Albumin (BSA, Sigma). Lymphocytes were purified by Lymphoprep Ficoll gradient (Thermo Fisher) and contaminating red blood cells were lysed by ACK buffer (Thermo Fisher). Cells were frozen in Fetal Bovine Serum (FBS, Gibco) with 10% Dimethyl sulfoxide (DMSO, Sigma) prior to downstream analysis. On the day of sorting, B cells were enriched using the human pan B cell EasySep™ enrichment kit (STEMCELL).

### Recombinant proteins and probe generation

SARS-CoV-2 proteins were obtained from the Krammer laboratory at Mt. Sinai, the Joachimiak laboratory at Argonne, and the Fremont laboratory at Washington University. pCAGGS expression constructs for the spike protein and spike RBD were obtained from the Krammer lab at Mt. Sinai and produced in house in Expi293F suspension cells (Thermo Fisher). Sequences for the spike and RBD proteins as well as details regarding their expression and purification have been previously described^31,32^. Proteins were biotinylated for 2 hours on ice using EZ-Link™ Sulfo-NHS-Biotin, No-Weigh™ Format (Thermo Fisher) according to the manufacturer’s instructions, unless previously Avi-tagged and biotinylated (ORF7a and ORF8 proteins, Fremont laboratory). Truncated cDNAs encoding the Ig-like domains of ORF7a and ORF8 were inserted into the bacterial expression vector pET-21(a) in frame with a biotin ligase recognition sequence at the c-terminus (GLNDIFEAQKIEWHE). Soluble recombinant proteins were produced as described previously^33^. In brief, inclusion body proteins were washed, denatured, reduced, and then renatured by rapid dilution following standard methods^34^. The refolding buffer consisted of 400 mM arginine, 100 mM Tris-HCl, 2 mM EDTA, 200 µM ABESF, 5 mM reduced glutathione, and 500 µM oxidized glutathione at a final pH of 8.3. After 24 hours, the soluble-refolded protein was collected over a 10 kDa ultrafiltration disc (EMD Millipore, PLGC07610) in a stirred cell concentrator and subjected to chromatography on a HiLoad 26/60 Superdex S75 column (GE Healthcare). Site-specific biotinylation with BirA enzyme was done following the manufacture’s protocol (Avidity) except that the reaction buffer consisted of 100mM Tris-HCl (pH 7.5) 150 mM NaCl, with 5 mM MgCl2 in place of 0.5 M Bicine at pH 8.3. Unreacted biotin was removed by passage through a 7K MWCO desalting column (Zeba spin, Thermo Fisher). Full-length SARS-CoV-2 NP was cloned into pET21a with a hexahistidine tag and expressed using BL21(DE3)-RIL *E. coli* in Terrific Broth (bioWORLD). Following overnight induction at 25°C, cells were lysed in 20 mM Tris-HCl pH 8.5, 1 M NaCl, 5 mM β-mercaptoethanol, and 5 mM imidazole for nickel-affinity purification and size exclusion chromatography. Biotinylated proteins were then conjugated to Biolegend TotalSeq™ PE streptavidin-(PE-SA) oligos at a 0.72:1 molar ratio of antigen to PE-SA. The amount of antigen was chosen based on a fixed amount of 0.5 µg PE-SA and diluted in a final volume of 10 µL. PE-SA was then added gradually to 10 µl biotinylated proteins 5 times on ice, 1 µl PE-SA (0.1 mg/ml stock) every 20 minutes for a total of 5 µl (0.5 µg) PE-SA. The reaction was then quenched with 5 µl 4mM Pierce™ biotin (Thermo Fisher) for 30 minutes for a total probe volume of 20 µL. Probes were then used immediately for staining.

### Antigen-specific B cell sorting

PBMCs were thawed and B cells were enriched using EasySep™ pan B cell magnetic enrichment kit (STEMCELL). B cells were stained with a panel containing CD19 PE-Cy7 (Biolegend), IgM APC (Southern Biotech), CD27 BV605 (Biolegend), CD38 BB515 (BD Biosciences), and CD3 BV510 (BD Biosciences). B cells were stained with surface stain master mix and each COVID-19 antigen probe for 30 minutes on ice in 1X PBS supplemented with 0.2% BSA and 2 mM Pierce Biotin. Cells were stained with probe at a 1:100 dilution (NP, ORF7a, ORF8, RBD) or 1:200 dilution (spike). Cells were subsequently washed with 1X PBS 0.2% BSA and stained with Live/Dead BV510 (Thermo Fisher) in 1X PBS for 15 minutes. Cells were washed again and re-suspended at a maximum of 4 million cells/mL in 1X PBS supplemented with 0.2% BSA and 2 mM Pierce Biotin for downstream cell sorting using the MACSQuantTyto cartridge sorting platform (Miltenyi). Cells that were viable/CD19^+^/antigen-PE^+^ were sorted as probe positive. The PE^+^ gate was drawn by use of FMO controls. Cells were then collected from the cartridge sorting chamber and used for downstream 10X Genomics analysis.

### 10X Genomics library construction

VDJ, 5’, and probe feature libraries were prepared using the 10X Chromium System (10X Genomics, Pleasanton, CA). The Chromium Single Cell 5’ Library and Gel Bead v2 Kit, Human B Cell V(D)J Enrichment Kit, and Feature Barcode Library Kit were used. All steps were followed as listed in the manufacturer’s instructions. Specifically, user guide CG000186 Rev D was used. Final libraries were pooled and sequenced using the NextSeq550 (Illumina, San Diego, CA) with 26 cycles apportioned for read 1, 8 cycles for the i7 index, and 134 cycles for read 2.

### Computational analyses for single cell sequencing data

We adopted Cell Ranger (version 3.0.2) for raw sequencing processing, including 5’ gene expression analysis, antigen probe analysis, and immunoprofiling analysis of B cells. Based on Cell Ranger output, we performed downstream analysis using Seurat (version 3.2.0, an R package, for transcriptome, cell surface protein and antigen probe analysis) and IgBlast (version 1.15, for immunoglobulin gene analysis). For transcriptome analysis, Seurat was used for cell quality control, data normalization, data scaling, dimension reduction (both linear and non-linear), clustering, differential expression analysis, batch effects correction, and data visualization. Unwanted cells were removed according to the number of detectable genes (number of genes <200 or >2500 were removed) and percentage of mitochondrial genes for each cell. A soft threshold of percentage of mitochondrial genes was set to the 95^th^ percentile of the current dataset distribution, and the soft threshold was subject to a sealing point of 10% as the maximum threshold in the case of particularly poor cell quality. Transcriptome data were normalized by a log-transform function with a scaling factor of 10,000 whereas cell surface protein and antigen probe were normalized by a centered log-ratio (CLR) normalization. We used variable genes in principal component analysis (PCA) and used the top 15 principal components (PCs) in non-linear dimension reduction and clustering. High-quality cells were then clustered by Louvain algorithm implemented in Seurat under the resolution of 0.6. Differentially expressed genes for each cell cluster were identified using a Wilcoxon rank-sum test implemented in Seurat. Batch effects correction analysis was performed using an Anchor method implemented in Seurat to remove batch effects across different datasets. All computational analyses were performed in R (version 3.6.3).

### Trajectory and pseudotime analyses

Trajectory analyses were performed using Monocle 3 (version 0.2.2)^35,36^, Seurat 3, and the SeuratWrappers package (version 0.2.0)^37^. Cells from multiple subjects were integrated to remove batch effects using Seurat, and all cells were clustered into two non-connected partitions. We then performed trajectory analysis on the main partition containing the majority of the cells and clusters (clusters 0–11). Pseudotime analysis of cells was also inferred from this major partition using Monocle3. The root node of the pseudotime analysis was set to cluster 2, a naïve B cell subset with the lowest degree of VH gene SHM and CSR.

### Selection of antibodies for mAb synthesis

Representative antibodies from each subject were chosen for synthesis by choosing random samplings of B cells that bound to a given antigen probe with higher intensity relative to all other probes. B cells with varying ranges of probe-binding intensities were chosen for confirmation by ELISA. B cells binding to all probes in a polyreactive manner were also chosen and validated for polyreactivity by polyreactivity ELISA (see methods below).

### Monoclonal antibody generation

Immunoglobulin heavy and light chain genes were obtained by 10X Genomics VDJ sequencing analysis and monoclonal antibodies (mAbs) were synthesized by Integrated DNA Technologies. Cloning, transfection, and mAb purification have been previously described^38^. Briefly, sequences were cloned into human IgG1 expression vectors using Gibson assembly, and heavy and light genes were co-transfected into 293T cells (Thermo Fisher). Secreted mAbs were then purified from the supernatant using protein A agarose beads (Thermo Fisher).

### Enzyme-linked immunosorbent assay (ELISA)

High-protein binding microtiter plates (Costar) were coated with recombinant SARS-CoV-2 proteins at 2 µg/ml in 1X PBS overnight at 4°C. Plates were washed the next morning with 1X PBS 0.05% Tween and blocked with 1X PBS containing 20% fetal bovine serum (FBS) for 1 hour at 37°C. Antibodies were then serially diluted 1:3 starting at 10 µg/ml and incubated for 1 hour at 37°C. Horseradish peroxidase (HRP)-conjugated goat anti-human IgG antibody diluted 1:1000 (Jackson Immuno Research) was used to detect binding of mAbs, and plates were subsequently developed with Super Aquablue ELISA substrate (eBiosciences). Absorbance was measured at 405 nm on a microplate spectrophotometer (BioRad). To standardize the assays, control antibodies with known binding characteristics were included on each plate and the plates were developed when the absorbance of the control reached 3.0 OD_405_ units. All experiments were performed in duplicate 2–3 times.

### Polyreactivity ELISA

Polyreactivity ELISAs were performed as previously described^39,40^. High-protein binding microtiter plates (Costar) were coated with 10 µg/ml calf thymus dsDNA (Thermo Fisher), 2 µg/ml Salmonella enterica serovar Typhimurium flagellin (Invitrogen), 5 µg/ml human insulin (Sigma-Aldrich), 10 µg/ml KLH (Invitrogen), and 10 µg/ml Escherichia coli LPS (Sigma-Aldrich) in 1X PBS. Plates were coated with 10 µg/ml cardiolipin in 100% ethanol and allowed to dry overnight. Plates were washed with water and blocked with 1X PBS/0.05%Tween/1mM EDTA. MAbs were diluted 1 µg/ml in PBS and serially diluted 4-fold, and added to plates for 1.5 hours. Goat anti-human IgG-HRP (Jackson Immunoresearch) was diluted 1:2000 in PBS/0.05%Tween/1mM EDTA and added to plates for 1 hour. Plates were developed with Super Aquablue ELISA substrate (eBioscience) until the positive control mAb, 3H9^41^, reached an OD_405_ of 3. All experiments were performed in duplicate.

### Memory B cell stimulations and enzyme-linked immunospot assays (ELISpot)

MBC stimulations were performed on PBMCs collected from subjects in the convalescent cohort. To induce MBC differentiation into antibody secreting cells, 1x10^6^ PBMCs were stimulated with 10 ng/ml Lectin Pokeweed Mitogen (Sigma-Aldrich), 1/100,000 Protein A from *Staphylococcus aureus*, Cowan Strain (Sigma-Aldrich), and 6 µg/ml CpG (Invitrogen) in complete RPMI in an incubator at 37µC/5% CO_2_ for 5 days. After stimulation, cells were counted and added to ELISpot white polystyrene plates (Thermo Fisher) coated with 4 µg/ml of SARS-CoV-2 spike that were blocked with 200 µl of complete RPMI. ELISpot plates were incubated with cells for 16 hours overnight in an incubator at 37°C/5% CO_2_. After the overnight incubation, plates were washed and incubated with anti-IgG-biotin and/or anti-IgA-biotin (Mabtech) for 2 hours at room temperature. After secondary antibody incubation, plates were washed and incubated with streptavidin-alkaline phosphatase (Southern Biotech) for 2 hours at room temperature. Plates were washed and developed with NBT/BCIP (Thermo Fisher Scientific) for 2–10 minutes, and reactions were stopped by washing plates with distilled water and allowed to dry overnight before counting. Images were captured with Immunocapture 6.4 software (Cellular Technology Ltd.), and spots were manually counted.

### Neutralization assay

The SARS-CoV-2/UW-001/Human/2020/Wisconsin (UW-001) virus was isolated from a mild case in February 2020 and used to assess neutralization ability of mAbs. Virus (~500 plaque-forming units) was incubated with each mAb at a final concentration of 10 µg/ml. After a 30-minute incubation at 37°C, the virus/antibody mixture was used to inoculate Vero E6/TMPRSS2 cells seeded a day prior at 200,000 cells per well of a TC12 plate. After 30 minutes at 37°C, cells were washed three times to remove any unbound virus, and media containing antibody (10 µg/ml) was added back to each well. Two days after inoculation, cell culture supernatant was harvested and stored at −80°C until needed. A non-relevant Ebola virus GP mAb and PBS were used as controls.

To determine the amount of virus in the cell culture supernatant of each well, a standard plaque-forming assay was performed. Confluent Vero E6/TMPRSS2 cells in a TC12 plate were infected with supernatant (undiluted, 10-fold dilutions from 10^−1^ to 10^−5^) for 30 minutes at 37°C. After the incubation, cells were washed three times to remove unbound virus and 1.0% methylcellulose media was added over the cells. After an incubation of three days at 37°C, the cells were fixed and stained with crystal violet solution in order to count the number plaques at each dilution and determine virus concentration given as plaque-forming units (PFU)/ml. A stringent cutoff for neutralization was chosen as 100-fold greater neutralization relative to the negative control mAb.

### Statistical analysis

All statistical analyses were performed using Prism software (GraphPad Version 7.0). Sample sizes (n) are indicated directly in the figures or in the corresponding figure legends and specific tests for statistical significance used are indicated in the corresponding figure legends. P values less than or equal to 0.05 were considered significant. *p<0.05, **p<0.01, ***p<0.001, ****p<0.0001.

## Extended Data

## Supplementary Material

Supplement **Extended Data Table 1.** Individual patient information.
**Extended Data Table 2.** Distribution of clinical parameters for patients included in the study.
**Extended Data Table 3.** MAbs generated from single B cell heavy and light chain gene sequences.
**Extended Data Table 4.** Public B cell clones identified from the integrated single cell sequencing dataset.

## Data Availability

The single B cell dataset generated during this study is available from the corresponding author on reasonable request or upon publication.
