# Supplementary material for: Distinct B cell subsets give rise to antigen-specific antibody responses against SARS-CoV-2
Source: Res Sq. 2020 Sep 25:rs.3.rs-80476. Preprint. [Version 1] doi: 10.21203/rs.3.rs-80476/v1 (PMC7523131; doi:10.21203/rs.3.rs-80476/v1)
Supplement: Supplement — Extended Data Table 1. Individual patient information. Extended Data Table 2. Distribution of clinical parameters for patients included in the study. Extended Data Table 3. MAbs generated from single B cell heavy and light chain gene sequences. Extended Data Table 4. Public B cell clones identified from the integrated single cell sequencing dataset. [file supplement_1.docx]

| **Subject ID** | **Age** | **Sex** | **Reported symptoms*** | **Duration of symptoms (days)** | **Symptom start to**  **donation (days)** | **Available data** |
| --- | --- | --- | --- | --- | --- | --- |
| 24 | 34 | M | Fatigue, cough, SOB, SC, fever, headache, BAP, diarrhea, LOS, LOT | 12 | 41 | Single cell probe binding, ELISPOT, serology |
| 20 | 31 | M | Fatigue, cough, SOB, SC, fever, headache, BAP, LOS, LOT | 19 | 48 | Single cell probe binding, ELISPOT, serology |
| 564 | 24 | F | Fatigue, cough, SOB, SC, ST, fever, headache, BAP, diarrhea, LOS, LOT | 32 | 60 | Single cell probe binding, ELISPOT, serology |
| 144 | 56 | M | Fatigue, cough, SC, ST, headache, BAP, LOS | 23 | 54 | Single cell probe binding, ELISPOT, serology |
| 214 | 47 | M | Fatigue, cough, SOB, SC, ST, headache, BAP, LOS, LOT | 24 | 59 | Single cell probe binding, ELISPOT, serology |
| 171 | 37 | F | Fatigue, cough, SOB, SC, fever, headache, BAP, diarrhea, LOS, LOT | 16 | 44 | Single cell probe binding, ELISPOT, serology |
| 92 | 35 | M | Fatigue, cough, SC, ST, fever, headache, BAP | 16 | 47 | Single cell probe binding, ELISPOT, serology |
| 48 | 45 | F | Fatigue, cough, SOB, SC, ST, fever, headache, AP, diarrhea, LOS, LOT | 8 | 40 | Single cell probe binding, ELISPOT, serology |
| 537 | 36 | M | Fatigue, cough, fever, BAP | 14 | 59 | Single cell probe binding, ELISPOT, serology |
| 586 | 32 | F | Fatigue, cough, SOB, SC, headache, BAP, AP, diarrhea | 17 | 61 | Single cell probe binding, ELISPOT, serology |
| 210 | 47 | M | Fatigue, cough, SOB, fever, headache, BAP, LOS, LOT | 7 | 41 | Single cell probe binding, ELISPOT, serology |
| 376 | 36 | F | Diarrhea, LOS, LOT | 7 | 48 | Single cell probe binding, ELISPOT, serology |
| 305 | 43 | F | Fatigue, cough, SC, ST, fever, headache, BAP, LOS, LOT | 4 | 47 | Single cell probe binding, ELISPOT, serology |
| 116 | 65 | F | Cough, SOB, fever, LOS, LOT | 18 | 49 | Single cell probe binding, ELISPOT, serology |
| 166 | 42 | F | Fatigue, cough, SOB, SC, fever, headache, BAP, diarrhea, LOS, LOT | 17 | 55 | Single cell probe binding, ELISPOT, serology |
| 155 | 47 | F | Fatigue, cough, SOB, ST, fever, BAP, LOS, LOT | 29 | 64 | Single cell probe binding, serology |
| 609 | 26 | F | Fatigue, SOB, ST, fever, headache, BAP, LOS, LOT | 7 | 57 | Single cell probe binding, serology |
| 282 | 34 | F | Fatigue, cough, SOB, fever, BAP, AP, LOS | 24 | 54 | ELISPOT, serology |
| 326 | 36 | F | Fatigue, cough, SC, fever, headache, BAP, AP, LOS, LOT | 15 | 47 | ELISPOT, serology |
| 356 | 51 | F | Fatigue, cough, ST, fever, headache, BAP, AP, diarrhea, LOS, LOT | 14 | 43 | ELISPOT, serology |
| 373 | 48 | M | Fatigue, fever, headache, BAP | 7 | 39 | ELISPOT, serology |
| 402 | 32 | F | Fatigue, cough, SOB, fever, headache, BAP, AP, diarrhea, LOS, LOT | 11 | 44 | ELISPOT, serology |
| 65 | 40 | F | Fatigue, SC, fever, headache, BAP, diarrhea, LOS, LOT | 13 | 47 | ELISPOT, serology |
| 423 | 58 | M | Fatigue | 5 | 38 | ELISPOT, serology |
| 558 | 56 | F | Fatigue, cough, SOB, LOS | 11 | 46 | ELISPOT, serology |

**Extended Data Table 1.** Individual patient information.

*SOB = shortness of breath; SC = sinus congestion; ST = sore throat; BAP = body aches and pain; AP = abdominal pain; LOS = loss of smell; LOT = loss of taste

| Median Age | 40 |
| --- | --- |
| Mean Age | 42 |
| Mode Age | 47 |
| Range Age | 24–65 |
| Number of Males | 9 |
| Number of Females | 16 |
| Median Duration of Symptoms (days) | 14 |
| Mean Duration of Symptoms (days) | 15 |
| Mode Duration of Symptoms (days) | 7 |
| Range Duration of Symptoms (days) | 4-32 |
| Median symptom start to donation (days) | 47 |
| Mean symptom start to donation (days) | 49 |
| Mode symptom start to donation (days) | 47 |
| Range symptom start to donation (days) | 38-64 |

**Extended Data Table 2.** Distribution of clinical parameters for patients included in the study.

| **mAb ID** | **Specificity** | | **Cluster** | **Isotype** | **# HC SHM** | **VH Gene** | **#LC SHM** | **Vk/L gene** |
| --- | --- | --- | --- | --- | --- | --- | --- | --- |
| S20-15 | Spike/RBD | | 7 | IgG1 | 8 | VH 4-59 | 1 | VL 3-21 |
| S20-22 | NP | | 9 | IgG1 | 7 | VH 4-4 | 4 | Vk 4-1 |
| S20-31 | NP | | 7 | IgG4 | 30 | VH 1-24 | 22 | Vk 3-20 |
| S20-40 | NP | | 2 | IgM | 0 | VH 4-4 | 1 | VL 2-14 |
| S20-58 | Spike/RBD | | 4 | IgG1 | 5 | VH 4-30 | 2 | Vk 2-24 |
| S20-74 | Spike/RBD | | 4 | IgG1 | 6 | VH 4-59 | 3 | VL 2-8 |
| S20-86 | Spike | | 7 | IgG1 | 9 | VH 3-9 | 2 | VL 2-14 |
| S24-68 | ORF8 | | 7 | IgG1 | 4 | VH 4-59 | 3 | VL 1-44 |
| S24-105 | ORF8 | | 7 | IgG1 | 6 | VH 3-48 | 4 | Vk 3-20 |
| S24-178 | NP | | 4 | IgG1 | 2 | VH 3-33 | 7 | VL 2-14 |
| S24-188 | NP | | 7 | IgG3 | 2 | VH 1-69 | 3 | VL 2-14 |
| S24-202 | NP | | 4 | IgG1 | 3 | VH 5-10 | 6 | Vk 3-11 |
| S24-278 | ORF8 | | 7 | IgG1 | 3 | VH 1-2 | 1 | Vk 3-20 |
| S24-339 | Spike/RBD | | 4 | Unknown | 5 | VH 3-49 | 1 | Vk 3-15 |
| S24-472 | ORF8 | | 7 | IgG1 | 5 | VH 4-4 | 4 | VL 4-16 |
| S24-490 | ORF8 | | 4 | IgM | 2 | VH 1-46 | 4 | Vk 3-20 |
| S24-494 | Spike/RBD | | 6 | IgG3 | 0 | VH 4-39 | 0 | Vk 1-39 |
| S24-566 | ORF8 | | 4 | IgG1 | 3 | VH 3-49 | 1 | Vk 2-28 |
| S24-636 | ORF8 | | 1 | IgD | 1 | VH 3-7 | 4 | VL 8-61 |
| S24-740 | ORF8 | | 7 | IgG1 | 5 | VH 1-3 | 1 | Vk 4-1 |
| S24-791 | NP | | 7 | IgG1 | 4 | VH 4-59 | 6 | Vk 3-20 |
| S24-902 | Spike/RBD | | 5 | IgG1 | 0 | VH 1-69 | 0 | VL 7-46 |
| S24-921 | NP | | 7 | IgG1 | 8 | VH 4-59 | 7 | Vk 1-39 |
| S24-1063 | NP | | 4 | IgG1 | 3 | VH 4-59 | 1 | Vk 3-20 |
| S24-1224 | Spike/RBD | | 4 | IgG1 | 7 | VH 1-46 | 7 | VL 1-40 |
| S24-1271 | Spike/RBD | | 7 | IgM | 6 | VH 3-66 | 6 | VL 3-1 |
| S24-1339 | Spike/RBD | | 4 | IgG1 | 1 | VH 3-53 | 1 | Vk 3-20 |
| S24-1345 | ORF8 | | 1 | IgD | 0 | VH 4-39 | 0 | Vk 1-13 |
| S24-1378 | ORF8 | | 1 | IgM | 0 | VH 3-53 | 0 | VL 8-61 |
| S24-1379 | NP | | 1 | IgG1 | 0 | VH 4-59 | 0 | VL 1-47 |
| S24-1384 | Spike/RBD | | 7 | IgG1 | 2 | VH 3-48 | 4 | VL 3-21 |
| S24-1476 | Spike/RBD | | 4 | IgG | 2 | VH 3-49 | 0 | Vk 3-15 |
| S24-1564 | NP | | 4 | IgG1 | 10 | VH 4-59 | 4 | Vk 1-39 |
| S24-1636 | NP | | 4 | IgG1 | 3 | VH 3-33 | 0 | Vk 3-11 |
| S24-1002 | Spike/RBD | | 7 | IgM | 3 | VH 3-30 | 5 | Vk 1-13 |
| S24-1301 | Spike | | 7 | IgG1 | 4 | VH 1-24 | 4 | VL 10-54 |
| S24-223 | Spike/RBD | | 4 | IgM | 1 | VH 2-5 | 3 | VL 2-14 |
| S24-461 | Spike/RBD | | 4 | IgG1 | 7 | VH 4-59 | 3 | VL 3-16 |
| S24-511 | NP | | 5 | IgD | 0 | VH 3-30 | 0 | VL 3-1 |
| S24-788 | Spike/RBD | | 5 | IgM | 0 | VH 3-33 | 1 | VL 3-1 |
| S24-821 | Spike/RBD | | 4 | IgM | 4 | VH2-70 | 0 | Vk 1-5 |
| S144-67 | Spike/RBD | | 7 | IgG1 | 7 | VH 5-51 | 5 | VL 1-40 |
| S144-69 | Spike/RBD | | 4 | IgG1 | 2 | VH 5-51 | 3 | Vk 1-5 |
| S144-94 | ORF8 | | 7 | IgG3 | 11 | VH 3-30 | 0 | Vk 2-28 |
| S144-113 | ORF8 | | 7 | IgG1 | 9 | VH 3-23 | 6 | Vk 1-39 |
| S144-175 | | ORF8 | 7 | IgG1 | 9 | VH 1-2 | 1 | VL 1-47 |
| S144-208 | | ORF8 | 4 | IgG1 | 6 | VH 1-2 | 7 | VL 2-11 |
| S144-339 | | NP | 4 | IgG1 | 11 | VH 3-21 | 7 | VK 3-20 |
| S144-359 | | ORF8 | 4 | IgG3 | 5 | VH 3-23 | 5 | Vk 1-39 |
| S144-460 | | Spike/RBD | 3 | IgA1 | 34 | VH 3-15 | 24 | Vk1D-17 |
| S144-466 | | Spike/RBD | 7 | IgG3 | 6 | VH 5-51 | 6 | Vk 1-5 |
| S144-469 | | ORF8 | 4 | IgG1 | 3 | VH 4-59 | 2 | Vk 2-28 |
| S144-509 | | Spike/RBD | 7 | IgG1 | 3 | VH 5-51 | 1 | Vk 1-5 |
| S144-516 | | ORF8 | 7 | IgG1 | 5 | VH 1-2 | 7 | VL 1-40 |
| S144-568 | | Spike/RBD | 6 | IgA2 | 11 | VH 4-59 | 11 | Vk 3-20 |
| S144-576 | | Spike/RBD | 4 | IgG1 | 3 | VH 1-69 | 2 | Vk 1-5 |
| S144-588 | | ORF8 | 7 | IgG1 | 1 | VH 4-39 | 3 | VL 3-1 |
| S144-628 | | Spike/RBD | 5 | IgA1 | 9 | VH 5-51 | 10 | VL 1-40 |
| S144-740 | | ORF8 | 7 | IgG1 | 1 | VH 1-2 | 5 | Vk 3-20 |
| S144-741 | | ORF8 | 4 | IgG1 | 5 | VH 1-2 | 1 | VL 1-44 |
| S144-803 | | Spike/RBD | 4 | IgG1 | 5 | VH 5-51 | 3 | Vk 1-5 |
| S144-843 | | ORF8 | 5 | Unknown | 20 | VH 3-30 | 8 | Vk 3-20 |
| S144-877 | | Spike/RBD | 7 | IgG1 | 2 | VH 3-30 | 6 | Vk 1-33 |
| S144-952 | | NP | 4 | IgM | 4 | VH 1-18 | 2 | Vk 4-1 |
| S144-971 | | ORF8 | 7 | IgG1 | 6 | VH 3-64 | 3 | Vk 4-1 |
| S144-1036 | | NP | 7 | IgG1 | 2 | VH 4-34 | 5 | Vk 4-1 |
| S144-1079 | | Spike/RBD | 7 | IgG1 | 7 | VH 1-69 | 3 | Vk 3-20 |
| S144-1299 | | ORF8 | 4 | IgG1 | 5 | VH 4-59 | 0 | VL 1-47 |
| S144-1339 | | Spike/RBD | 4 | IgG1 | 12 | VH 1-2 | 5 | VL 2-14 |
| S144-1406 | | Spike/RBD | 7 | IgG2 | 3 | VH 1-3 | 0 | Vk 1-5 |
| S144-1407 | | Spike/RBD | 4 | IgG1 | 6 | VH 1-69 | 2 | Vk 1-5 |
| S144-1569 | | ORF8 | 7 | IgG1 | 7 | VH 1-18 | 1 | VL 9-49 |
| S144-1641 | | Spike/RBD | 7 | IgG1 | 4 | VH 5-51 | 9 | Vk 1-5 |
| S144-1827 | | Spike/RBD | 3 | IgM | 20 | VH 3-7 | 5 | Vk 3-20 |
| S144-1848 | | NP | 7 | IgG1 | 4 | VH 3-21 | 8 | VL 1-47 |
| S144-1850 | | Spike/RBD | 7 | IgG1 | 2 | VH 3-23 | 3 | Vk 1-5 |
| S144-2234 | | ORF8 | 4 | IgG1 | 4 | VH 1-69 | 3 | Vk 4-1 |
| S564-105 | | NP | 4 | IgG1 | 5 | VH 4-61 | 2 | VL 2-14 |
| S564-14 | | Spike/RBD | 4 | IgD | 3 | VH 3-7 | 0 | Vk 3-21 |
| S564-68 | | Spike/RBD | 7 | IgG1 | 6 | VH 1-2 | 2 | VL 2-8 |
| S564-98 | | NP | 7 | IgG3 | 0 | VH 4-59 | 3 | Vk 1-39 |
| S564-105 | | NP | 4 | IgG1 | 5 | VH 4-61 | 2 | VL 2-14 |
| S564-134 | | Spike/RBD | 7 | IgG1 | 2 | VH 1-2 | 6 | VL 2-8 |
| S564-138 | | Spike/RBD | 4 | IgG1 | 8 | VH 1-2 | 1 | VL 2-14 |
| S564-152 | | Spike/RBD | 7 | IgG1 | 4 | VH 3-33 | 4 | Vk 1-33 |
| S564-218 | | Spike/RBD | 5 | IgM | 1 | VH 1-69 | 0 | VL 2-8 |
| S564-249 | | NP | 4 | IgA1 | 19 | VH 3-64 | 19 | VL 2-14 |
| S564-265 | | Spike/RBD | 7 | IgG1 | 4 | VH 1-2 | 3 | VL 2-8 |
| S564-275 | | NP | 4 | IgM | 3 | VH 4-59 | 6 | Vk 1-39 |
| S564-287 | | ORF8 | 4 | IgM | 1 | VH 1-2 | 3 | VL2-14 |

**Extended Data Table 3.** MAbs generated from single B cell heavy and light chain gene sequences.

| **B cell clone** | **Clonal Pool** | **Antigen** | **VH gene** | **DH gene** | **JH gene** | **LC V gene** | **LC J gene** | **HC CDR3 AA sequence** | **LC CDR3 AA sequence** | **Cluster** |
| --- | --- | --- | --- | --- | --- | --- | --- | --- | --- | --- |
| S144-121 | 1 | Spike | 3-23*01 | N/A | 4*02 | k3-20*01 | k1*01 | AKGSSTARPYYFDY | QEYGSSRM | 5 |
| S155-37 | 1 | Spike | 3-23*01 | 6-13*01 | 4*02 | k3-20*01 | k1*01 | VKGSAAARPYYFDY | QQYGNSRI | 3 |
| S210-896 | 2 | Spike | 3-30-3*01 | 1-7*01 | 4*02 | k3-20*01 | k3*01 | ARGHGNYLTYFDY | QQYGSSPLT | 5 |
| S376-2486 | 2 | Spike | 3-30-3*01 | 1-26*01 | 4*02 | k3-20*01 | k4*01 | ARGRGNYFTYFDY | QQYGGSLT | 7 |
| S166-2620 | 3 | Spike | 3-7*03 | 6-19*01 | 4*02 | l3-1*01 | l2*01 | ARDSIAVAGGLDY | QAWDSSTVV | 5 |
| S166-1318 | 3 | Spike | 3-7*03 | 6-19*01 | 4*02 | l3-1*01 | l2*01 | ARDGIAVAGGFDY | QAWDSSTVV | 4 |
| S171-1150 | 3 | Spike | 3-7*01 | 6-19*01 | 4*02 | l3-1*01 | l2*01 | ARDGIAVAGGLDY | QAWDSSTVV | 9 |
| S210-852 | 3 | Spike | 3-7*01 | 6-19*01 | 4*02 | l3-1*01 | l2*01 | ARDGIAVAGGFDY | QAWDSSTSVV | 4 |
| S305-968 | 3 | Spike | 3-7*03 | 6-19*01 | 4*02 | l3-1*01 | l2*01 | ARDSIAVAGGFDY | QAWDSSTNVV | 5 |
| S564-128 | 4 | NP | 3-7*01 | 1-26*01 | 4*02 | k3-15*01 | k2*01 | ARGDGSNSGIYFDS | QQYNYWYT | 5 |
| S469-373 | 4 | NP | 3-7*03 | 6-6*01 | 4*02 | k3-15*01 | k2*01 | ARGGGSSSGLYFES | QQYNYWYT | 5 |
| S144-292 | 5 | Spike | 5-51*01 | 2-21*02 | 4*02 | k1-5*01 | k1*01 | ARLFCGGDCPFDY | QQYNTYPRT | 7 |
| S2141-65 | 5 | Spike | 5-51*01 | 2-21*02 | 4*02 | k1-5*01 | k1*01 | ARQFCGGDCPFDY | QQYNSYPRT | 8 |
| S144-1364 | 5 | Spike | 5-51*01 | 3-10*01 | 4*02 | k1-5*01 | k2*01 | ARPNYYGSGSPPGY | QQYNSYYT | 5 |
| S210-1139 | 5 | Spike | 5-51*01 | 3-10*01 | 4*02 | k3-20*01 | k1*01 | ARPFYYGSESPPGY | QLFGSSPTWT | 4 |

**Extended Data Table 4.** Public B cell clones identified from the integrated single cell sequencing dataset.
